# Characterization of the complete chloroplast genome of shrubby sophora (*Sophora flavescens* Ait.)

**DOI:** 10.1080/23802359.2018.1532839

**Published:** 2018-10-26

**Authors:** Wenlong Zhang, Li Li, Guohong Li

**Affiliations:** a Guizhou Key Laboratory of Miao Medicine, Guiyang University of Traditional Chinese Medicine, Guiyang, Guizhou, China;; b Department of Pharmacy, Guiyang University of Traditional Chinese Medicine, Guiyang, Guizhou, China;; c School of Life Sciences, Guizhou Normal University, Guiyang, Guizhou, China

**Keywords:** *Sophora flavescens*, chloroplast genome, Illumina sequencing, phylogenetic analysis

## Abstract

*Sophora flavescens*, with high medicinal value, is a traditional Chinese medical plant wildly distributed in China. In this study, the complete chloroplast (cp) genome of *Sophora flavescens* was determined through Illumina sequencing method. The complete chloroplast genome of *S. flavescens* was 154,378 bp in length and contained a pair of IR regions (28,876 bp) separated by a small single copy region (18,110 bp) and a large single copy region (84,516 bp). The cp genome of *S. flavescens* encoded 130 genes including 84 protein-coding genes, 37 tRNA genes and eight ribosomal RNA genes. The overall GC content of *S. flavescens* cp genome is 36.6%. By phylogenetic analysis using ML method, *S. flavescens* showed the closest relationship with *Sophora alopecuroides*.

The shrubby sophora (*Sophora flavescens*) is a species of plant in the genus *Sophora*, which belongs to the Fabaceae family. It is a traditional Chinese medicine that has been used for anti-tumour, viral hepatitis, enteritis, viral myocarditis, arrhythmia, and skin diseases (Sun et al. 2012). Due to its important medicinal value, lots of scientific researches were reported to clarify corresponding functions of secondary metabolites in the root of *Sophora flavescens.* While the DNA information derived from genome and organelle genomes of *S. flavescens* is still limited. Here, we assembled and analyzed the chloroplast genome of *S. flavescens* based on the next-generation sequencing method. Our aim was to retrieve valuable cp molecular markers, indels and SSRs by comparative analyses with other valuable *Sophora* species.

Plant materials of *Sophora flavescens* Ait. sequenced in this study were acquired from medical plants garden in Guiyang University of Traditional Chinese Medicine. Total genomic DNA for genome sequencing was extracted exclusively from fresh young leaves using the cetyltrimethylammonium bromide (CTAB) method and was stored at -20 °C in the Key Laboratory of Miao Medicine, Guiyang University of Traditional Chinese Medicine. For high-throughput sequencing (NGS), the paired-end library from DNA extracts was prepared with a NEBNext Library building kits, following manufacturer’s protocol. Then, the library was sequenced on an Illumina HiSeq2500 platform. After reads quality filtration, the clean reads were assembled by SPAdes 3.6.1 (Bankevich et al. 2012) with default settings. We used the chloroplast genome of *Sophora alopecuroides* (MF156140.1) as a reference sequence to align the contigs and identify gaps. To fill the gap, Price (Ruby et al. 2013) and MITObim v1.8 (Hahn et al. 2013) were applied and Bandage (Wick et al. 2015) was used to identify the borders of the IR, LSC, and SSC regions. The complete sequence was primarily annotated by Plann (Huang and Cronk 2015) combined with manual correction. All tRNAs were confirmed using the tRNAscan-SE search server (Lowe et al. 1997). Other protein-coding genes were verified by BLAST search on the NCBI website (http://blast.ncbi.nlm.nih.gov/), and manual correction for start and stop codons was conducted. The circular cp genome map was drawn using OrganellarGenomeDRAW (Lohse et al. 2007). This complete chloroplast genome sequence together with gene annotations were submitted to GenBank under the accession numbers of MH748034.

The chloroplast genome of *Sophora flavescens* Ait. is a typical quadripartite structure with a length of 154,378 bp. The whole cp genome contains a large single-copy (LSC) region of 84,516 bp, a small single-copy (SSC) region of 18,110 bp, and two inverted repeats (IRs) regions of 28,876 bp (Figure 1). The cp genome possesses 130 genes, including 84 protein-coding genes (78 PCG species), eight ribosomal RNA genes (four rRNA species) and 37 tRNA genes (30 tRNA species). The overall GC content of the cp genome is 36.6%. The genome structure, gene order and GC content are similar to those of *Sophora alopecuroides* cp genome.

**Figure 1.**
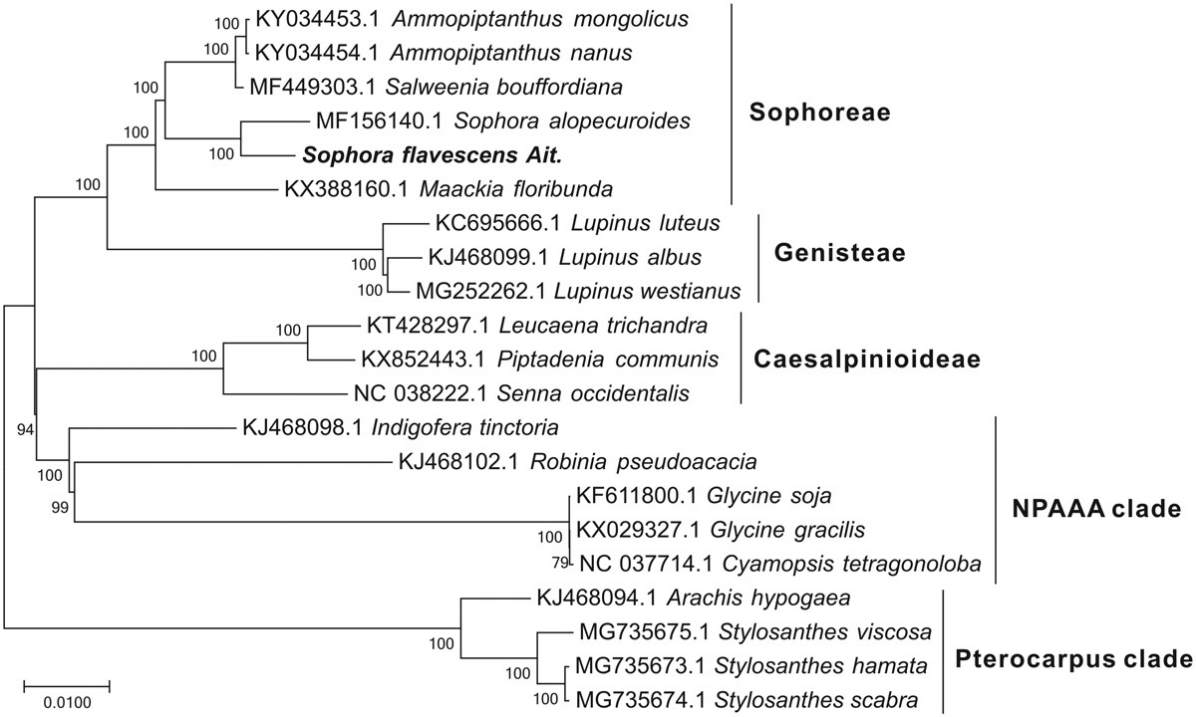
Phylogenetic tree yielded by ML analysis of 21 higher plant cp genomes. ML consensus tree is shown with bootstrap supports indicated by numbers besides branches.

For phylogenetic analysis assessing the relationship of this plastid, we selected other 20 higher plant cp genomes from Sophoreae (five taxa), Genisteae (three taxa), Caesalpinioideae (three taxa), NPAAA clade (five taxa) and the Pterocarpus clade (four taxa) to construct a genome-wide alignment. We considered plastids of the Pterocarpus clade as the outgroup. The genome-wide alignment of all cp genomes was done by HomBlocks (Bi et al. 2018), resulting in 69,597 positions in total. The whole genome alignment was analyzed by IQ-TREE version 1.6.6 (Nguyen et al. 2014) under the TIM3 + F + R3 model. The tree topology was verified under both 1000 bootstrap and 1000 replicates of SH-aLRT test. As shown in Figure 1, the phylogenetic positions of these 21 cp genomes were successfully resolved with full bootstrap supports across almost all nodes. *Sophora flavescens* Ait. belongs to the Sophoreae clade as expected, and exhibited the closest relationship with *Sophora alopecuroides*.
